# Antinuclear Antibody (ANA)‑Negative, Anti-melanoma Differentiation-Associated Gene 5 (MDA5)‑Positive Dermatomyositis Presenting as Rapidly Progressive Interstitial Lung Disease: A Case Report

**DOI:** 10.7759/cureus.114432

**Published:** 2026-08-12

**Authors:** Joy Nish, Annabel Crippen, Vijaya Ramalingam

**Affiliations:** 1 Internal Medicine, Northeast Georgia Medical Center, Gainesville, USA; 2 Internal Medicine, Edward Via College of Osteopathic Medicine, Spartanburg, USA; 3 Pulmonology and Critical Care Medicine, Northeast Georgia Medical Center, Gainesville, USA

**Keywords:** anti-mda-5, ild without myositis, interstitial lung disease (ild), new-onset ild, rapidly progressive ild

## Abstract

Dermatomyositis (DM) is a rare, autoimmune myopathy characterized by proximal muscle weakness and pathognomonic skin findings, including Gottron's papules and a heliotrope rash. Anti-melanoma differentiation-associated gene 5 (MDA5)-positive DM is a distinct subtype often associated with rapidly progressive interstitial lung disease (RP-ILD) and may lack the classic features of DM. We present the case of a 43-year-old woman who developed persistent hypoxic respiratory failure after multiple vascular surgeries requiring repeated postoperative blood transfusions. Her respiratory failure was initially attributed to transfusion-related acute lung injury with progression to acute respiratory distress syndrome. Despite appropriate treatment, her respiratory condition worsened. Infectious and autoimmune workups, including bronchoscopy and ANA testing, were unrevealing. Persistent diffuse bilateral ground-glass opacities and new fibrotic changes on imaging prompted an extended myositis panel, which revealed positive anti-MDA5 antibodies, establishing a diagnosis of RP-ILD secondary to MDA5 DM. She was treated with rituximab, mycophenolate, and IV immunoglobulin, with clinical improvement and was discharged on 4L of supplemental oxygen. This case underscores the importance of considering anti-MDA5-positive DM in unexplained, progressive respiratory failure, highlighting the importance of early recognition and multidisciplinary management.

## Introduction

Dermatomyositis (DM) is a rare, acquired autoimmune myopathy characterized by proximal muscle weakness and distinctive skin findings, with an estimated incidence rate of 9.63 per 1,000,000 individuals affected each year [1]. Although uncommon, its development has been linked to genetic susceptibility, with certain human leukocyte antigen types conferring an increased risk [2]. The pathophysiology of the disease is thought to involve a humoral autoantibody-mediated attack on muscle capillaries and arterial endothelium [3]. Pathognomonic skin manifestations include Gottron's papules and a heliotrope rash, which may precede or coincide with proximal muscle weakness. Beyond muscle and skin involvement, DM carries significant systemic associations. It has been associated with gastric and colorectal cancer and can also affect the heart and lungs [4]. Several autoantibodies define distinct DM phenotypes. Among them, anti-melanoma differentiation-associated gene 5 (MDA5)-positive DM represents a rare, but clinically important subset [3]. This form occurs more frequently in Asian populations and is characterized by minimal muscle involvement with a high risk of developing rapidly progressive interstitial lung disease (RP-ILD), a leading cause of morbidity and mortality in this group [5]. A patient meets the criteria for RP-ILD if, within one month of respiratory symptom onset, they have any of the following: worsening dyspnea requiring hospitalization or supplementary oxygen; a decline in forced vital capacity (FVC) of more than 10% or a decline in diffusion capacity for carbon monoxide of more than 15% with decreased FVC; CT imaging showing an increase in interstitial abnormalities by more than 20%, or arterial blood gas analysis indicating respiratory failure, such as a reduction in the partial pressure of oxygen (PaO_2_) partial pressure greater than 10 mmHg [6].

Diagnosis can be especially challenging because antinuclear antibody (ANA) testing, often the first autoimmune screening test ordered, is negative in up to 81% of MDA5-positive patients [6]. However, ANA negativity is not specific to this subtype. In a recent study of 320 individuals with classic and amyopathic DM, ANA titers were positive in only 63% of those with classic DM and 49% of those with amyopathic DM, corresponding to ANA-negativity rates of 37% and 51%, respectively [7]. Other studies have also reported that while ANA testing is highly sensitive, its low specificity limits its diagnostic utility in inflammatory myopathies [8]. Therefore, ordering a myositis panel is more informative in patients with high clinical suspicion, with studies demonstrating a sensitivity of 80% and a specificity of 75.76% [9].

The aggressive nature of RP-ILD contributes to its poor prognosis, underscoring the need for early recognition and prompt initiation of immunosuppressive therapy. Here, we report a case of ANA-negative, anti-MDA5-positive DM with RP-ILD occurring shortly after hospitalization for major vascular surgery. Given the patient's history of repeated postoperative blood transfusions, transfusion-related acute lung injury (TRALI) with progression to acute respiratory distress syndrome (ARDS) was initially considered in the differential diagnosis. However, the patient's persistent and atypical clinical course prompted further evaluation. This case highlights the importance of considering rare autoimmune myopathies in the differential diagnosis of patients with unexplained, rapidly progressive respiratory failure.

## Case presentation

A 43-year-old woman with a past medical history of aortobifemoral graft, tobacco abuse, and type 2 diabetes mellitus presented to the emergency department with a three-day history of right lower extremity pain, mottling, and blue discoloration. CTA of the aorta and abdomen with runoff confirmed complete occlusion of the graft extending to the bilateral common femoral arteries. The patient subsequently underwent urgent graft thrombectomy with bilateral iliac stents, requiring one unit of packed red blood cells (PRBCs).

The next day, the patient continued to report loss of motor and sensory function in the right lower extremity, prompting a return to the OR for a right axillary-bilateral femoral bypass with multilevel right lower extremity thrombectomies. She was subsequently transferred to the ICU for closer monitoring, where she shortly thereafter became hypotensive and tachycardic; 1 unit of PRBCs was transfused for a hemoglobin of 6.7. Three days later, she was transferred out of the ICU to the hospital medicine team, where she received additional units of PRBCs for persistently low hemoglobin.

Eleven days after her first surgery, she developed fever and shortness of breath. Chest X-ray was suggestive of pulmonary vascular congestion, and she was started on linezolid and cefepime for suspected multifocal pneumonia. The following day, she returned to the OR for an evacuation of a right infraclavicular hematoma identified on physical examination. During this procedure, she received an additional four units of PRBCs.

**Figure 1:**
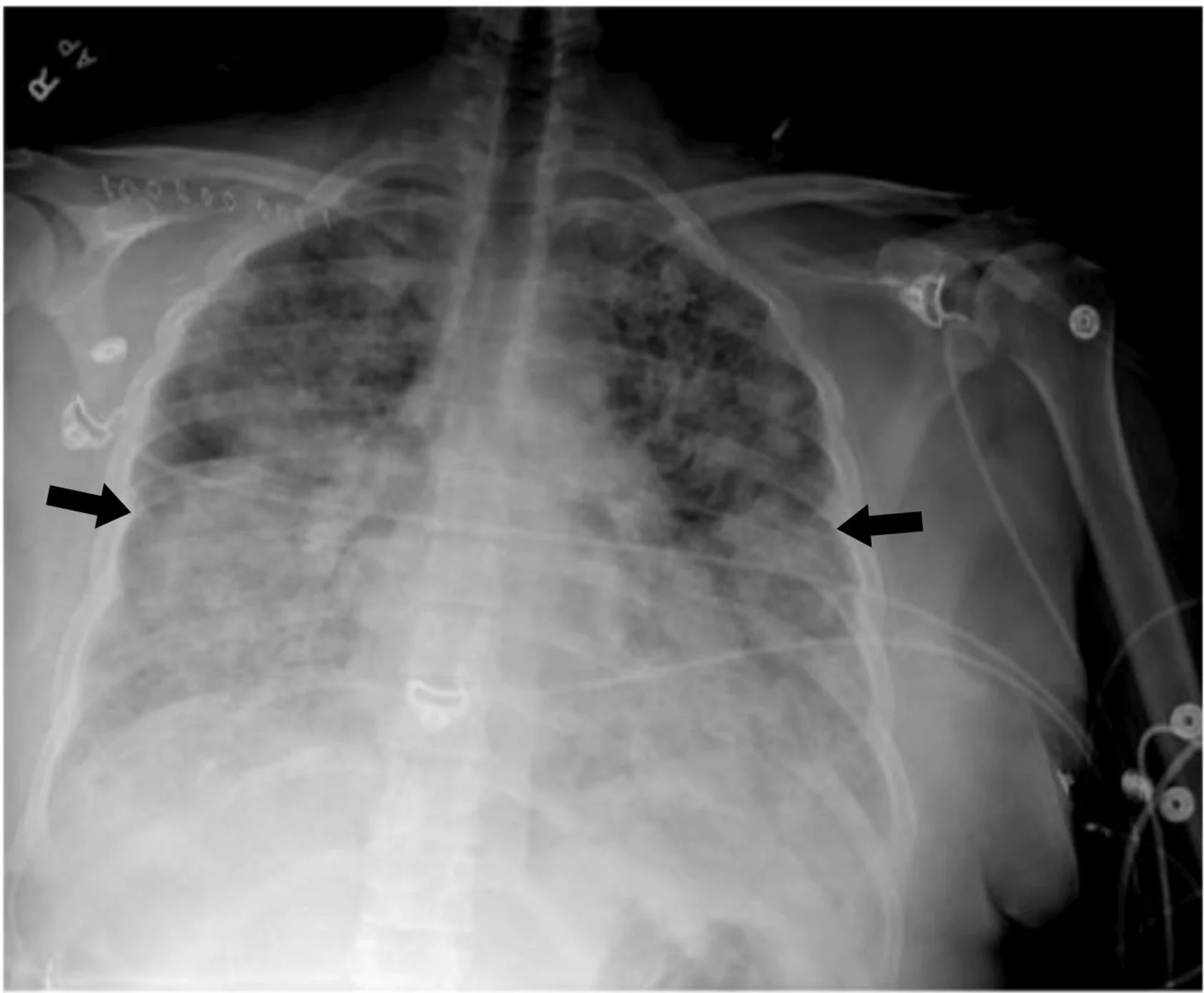
Chest X-ray demonstrating bilateral pulmonary opacities (black arrows) 14 days after patient's graft thrombectomy, status post eight units of packed red blood cells.

**Figure 2:**
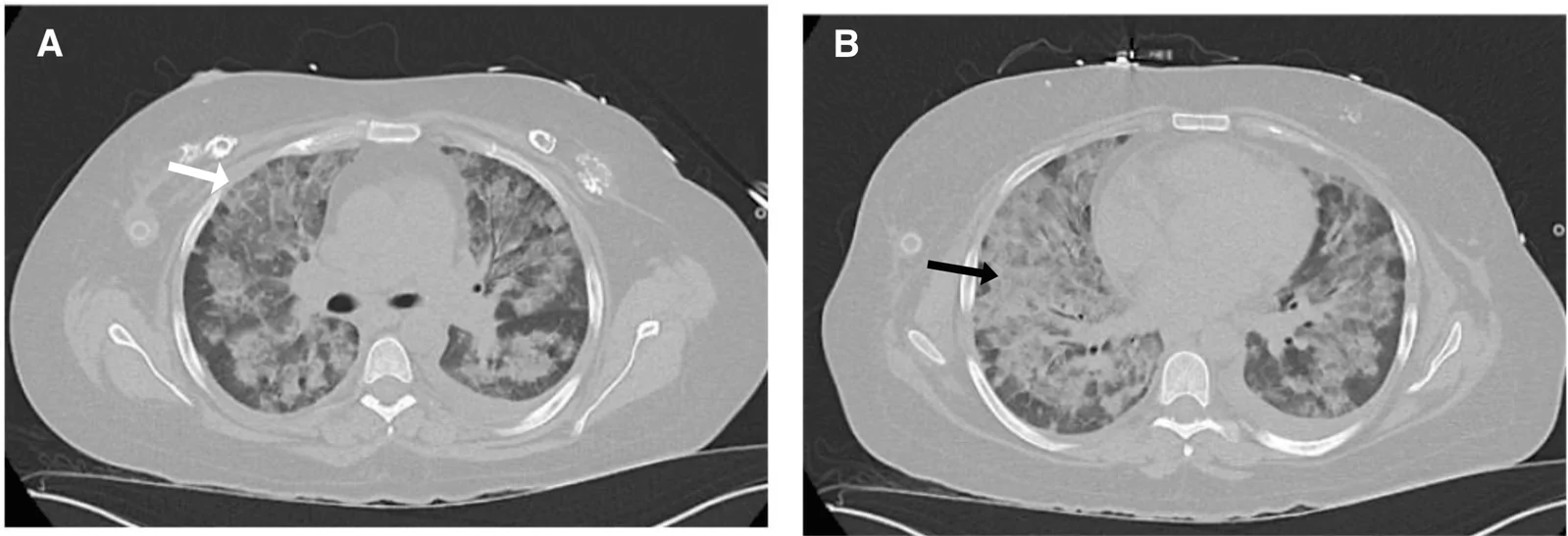
Non-contrast chest CT obtained one day after repeat chest radiography demonstrated (a) diffuse bilateral ground-glass (white arrow) and (b) consolidative changes (black arrow) throughout both lungs.

Despite broad-spectrum antibiotic treatment, her respiratory status continued to decline. Repeat chest X-ray revealed worsening bilateral pulmonary opacities (Figure 1), thought to represent TRALI or acute interstitial pneumonia. Given this new finding, she was started on methylprednisolone 40 mg daily. A non-contrast CT Chest performed the following day confirmed diffuse bilateral ground-glass and reticular infiltrates (Figure 2). Her methylprednisolone was then escalated to twice a day along with her ongoing antibiotics, linezolid and cefepime. However, before her respiratory condition fully resolved, the patient elected to leave the hospital against medical advice and was discharged on 4L of supplemental oxygen with a prednisone taper.

**Figure 3:**
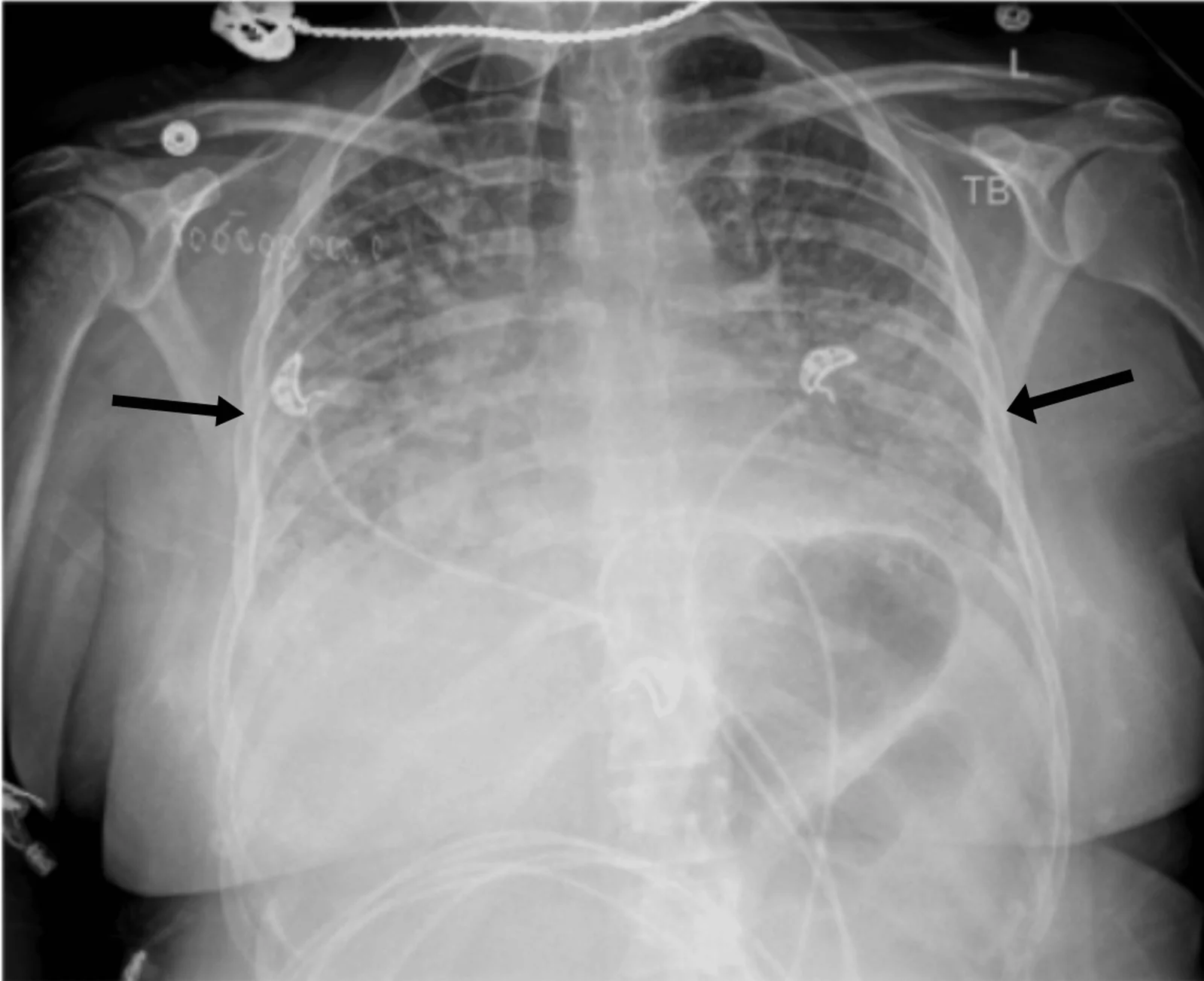
Chest X-ray demonstrating persistent, diffuse bilateral opacities (black arrow) at readmission, almost one month following patient's initial thrombectomy surgery.

**Figure 4:**
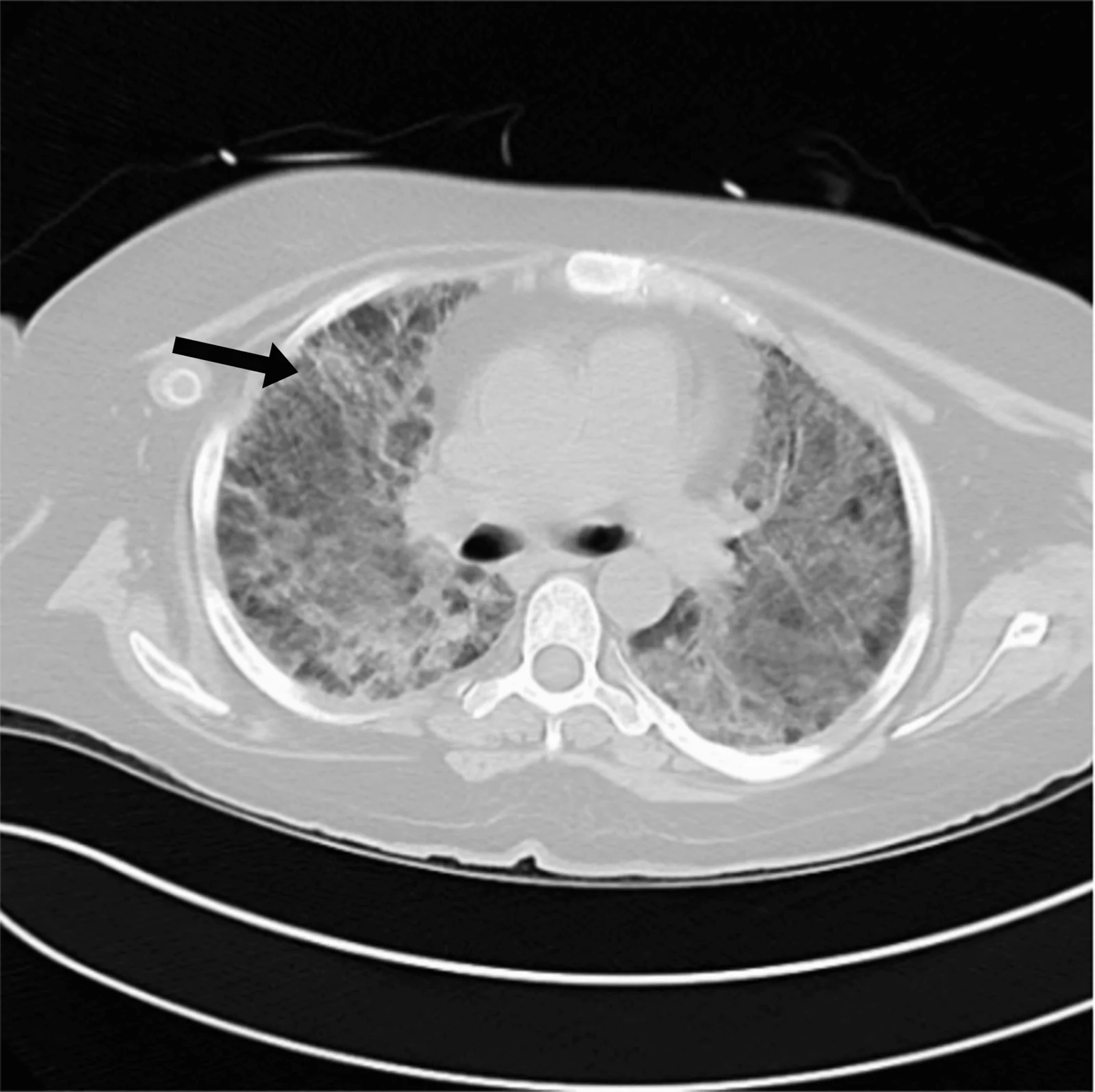
Non-contrast chest CT obtained eight days into readmission, demonstrating persistent, diffuse ground glass opacities (black arrow).

The patient returned to the ED three days later with worsening shortness of breath and was transferred to the ICU after being found to be in septic shock, requiring vasopressors and high-flow nasal cannula at 80-90% FiO2 and 45 L with alternative BiPAP support. A chest X-ray at the time of admission showed persistent bilateral pulmonary opacities (Figure 3). We restarted intravenous methylprednisolone 80 mg every eight hours, along with piperacillin/tazobactam, micafungin, and linezolid. The next day, her methylprednisolone dose was escalated to 125 mg every six hours for three days and then de-escalated back to 80 mg every eight hours. Due to a lack of clinical improvement over the next few days, a chest CT was repeated, which demonstrated diffuse bilateral ground-glass opacities (Figure 4). Further workup for other potential etiologies of her lung disease included negative results for HIV, vasculitis panel, PJP PCR, and ANA panel. Bronchoscopy with bronchoalveolar lavage was also negative for any microorganisms.

Over the next few weeks, her respiratory status gradually improved, but she remained dependent on high-flow nasal cannula despite treatment with intravenous corticosteroids. After five weeks of intravenous methylprednisolone, she was transitioned to an oral prednisone taper, which was gradually weaned over the following four weeks. Despite this, serial CT chest imaging continued to demonstrate persistent bilateral ground-glass opacities and fibrotic changes without significant interval improvement. Around this time, an extended myositis panel was sent for further evaluation of fibrotic lung disease and returned positive for MDA5-Ab, revealing a diagnosis of anti-MDA5 DM with rapidly RP-ILD. Additional serologic testing demonstrated a negative ANA, Jo-1 antibody of 2, and negative PM/Scl-100 antibody, with elevated CRP of 2.34 mg/dL (reference range, <0.6 mg/dL), CK of 519 U/L (reference range, 34-145 U/L), aldolase of 8.1 U/L (reference range, 1.2-7.6 U/L), and LDH of 700 U/L (reference range, 84-246 U/L). Other DM-specific antibodies tested, including anti-Mi-2, PL-7, PL-12, P155/140, EJ, Ku, SRP, OJ, anti-TIF1-γ, anti-NXP2, Ha, Ks, Zo, and anti-SAE, were negative.

**Figure 5:**
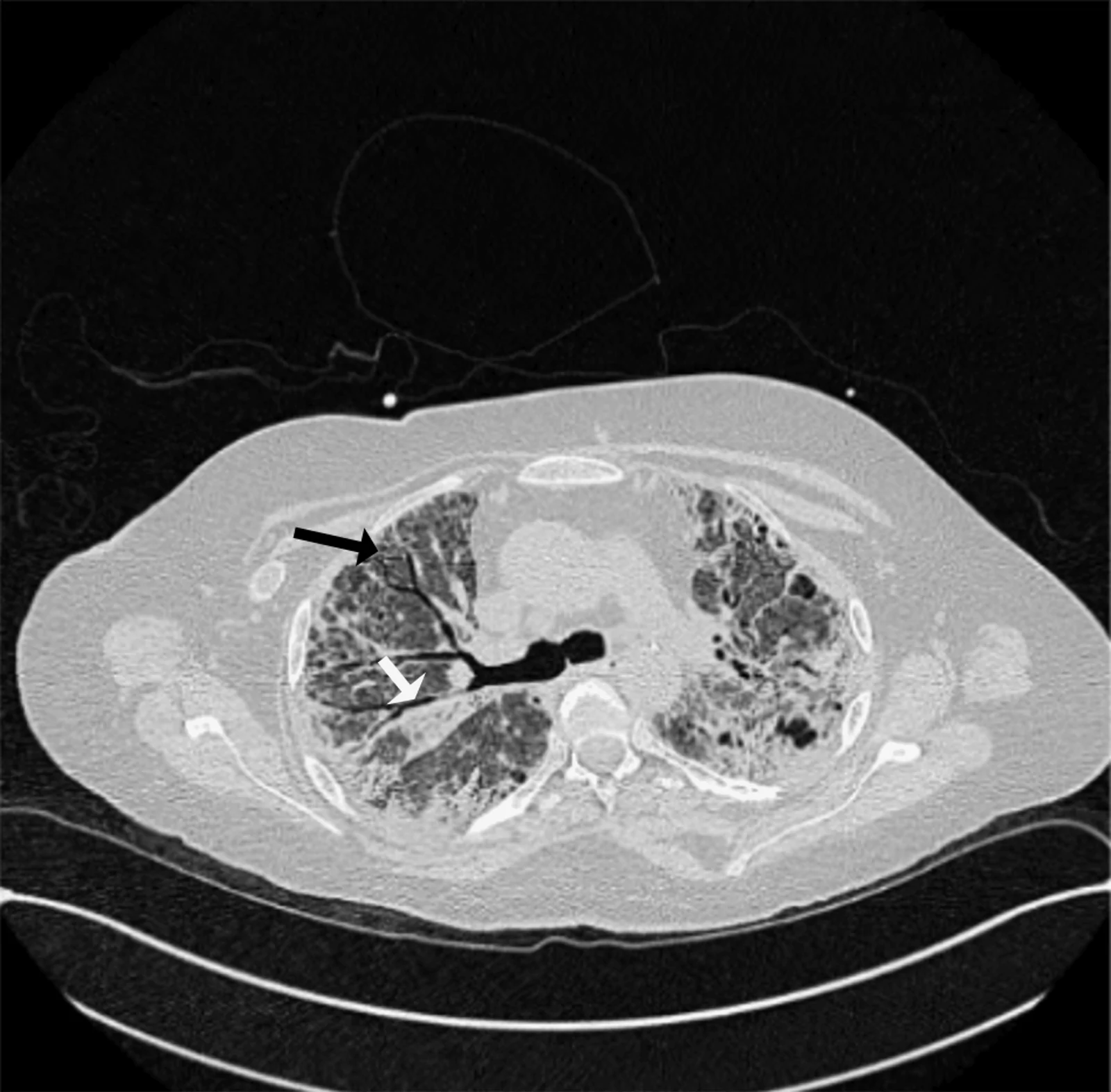
High-resolution chest CT obtained two weeks following initiation of immunosuppression therapy demonstrating fibrotic changes with improvement of previously noticed ground-glass opacities (black arrow) and traction bronchiectasis (white arrow).

On further interview, the patient reported prior rashes on her forehead, eyebrows, abdomen, and upper back, but no skin findings were present during her prolonged hospital course. Rheumatology was consulted, and she received two doses of rituximab, 2g/kg of IV immunoglobulin, and was started on mycophenolate. This treatment regimen was selected over alternatives such as cyclophosphamide or plasma exchange because her lung status had stabilized with steroids-suggesting a more subacute process-by the time immunosuppressive therapy was initiated, and there were concerns for adverse effects from the alternative options given her prolonged hospital course and co-morbidities. A high-resolution CT performed two weeks after treatment showed improvement of previously noticed GGO (Figure 5). Her supplemental oxygen was weaned to 4L, and she was discharged home with plans to follow up with pulmonary and rheumatology outpatient. Unfortunately, despite multiple attempts to reach the patient, she has been lost to follow-up, and we currently do not know of her respiratory status.

## Discussion

While classic DM presents with well-recognized cutaneous and muscular features, the MDA5-positive subtype often manifests with minimal muscle involvement and atypical or absent rashes [10]. Its strongest clinical association is with rapidly progressive ILD, a complication that carries high mortality and frequently precedes other recognizable features of DM [6]. This overlap with more common diagnoses such as ARDS or new-onset pneumonia creates significant diagnostic challenges, as illustrated in our patient.

Our patient’s presentation was particularly unique in that she developed severe hypoxemia following complex vascular surgery and multiple blood transfusions, initially attributed to TRALI progressing to ARDS. Early chest CT showed diffuse ground-glass opacities with reticular infiltrates. Despite antibiotics and supportive care, respiratory symptoms persisted. Serial imaging revealed ongoing ground-glass opacities and fibrotic changes, prompting evaluation for alternative etiologies. Given her history of recurrent arterial thrombosis at a young age despite anticoagulation, antiphospholipid antibody syndrome and vasculitis were ruled out. However, further autoimmune evaluation with a myositis panel returned positive for anti-MDA5 antibodies, confirming anti-MDA5-positive DM as the underlying cause of rapidly progressive ILD.

Although we do not have an anti-MDA5 antibody titer for our patient, the presence of anti-MDA5 antibodies is well established as being strongly associated with ILD, particularly the rapidly progressive phenotype that aligns with this patient’s clinical presentation [11]. Importantly, there is no universally standardized absolute anti-MDA5 antibody cutoff for diagnosis, as thresholds vary by assay platform, units, and laboratory methodology.

Most clinical laboratories use ELISA, which demonstrates excellent analytical performance, with reported sensitivity of 98.2% and specificity of 100% when compared with the gold-standard immunoprecipitation assay [12]. While absolute diagnostic cutoffs vary, prognostic thresholds have been more clearly defined. For ELISA-based assays, an anti-MDA5 IgG1 level >13 U/mL has been validated as an independent predictor of mortality in a large cohort study [13]. Similarly, for immunofluorescence-based assays, an anti-MDA5 IgG1 titer >1:100 has been associated with rapidly progressive ILD [14]. These findings support the clinical relevance of anti-MDA5 antibody positivity even in the absence of a specific titer value for this patient, particularly given the characteristic pulmonary manifestations observed.

This case underscores the need for diagnostic vigilance in patients with unexplained hypoxemia and no prior lung disease, particularly in middle-aged women, who are at increased risk for autoimmune conditions. In this patient, persistent oxygen requirements, non-resolving ground-glass opacities, and evolving fibrosis despite corticosteroid therapy prompted further evaluation, ultimately revealing anti-MDA5-positive DM as the underlying cause.

MDA5 is a cytosolic sensor of viral RNA that triggers type I interferon and proinflammatory cytokine responses. In autoimmune disease, sustained interferon signaling promotes lung injury and fibrosis via macrophage activation and TGF-β-mediated fibroblast remodeling [15]. This pathophysiology explains why anti-MDA5-positive DM patients may develop severe ILD without classic DM features. Although our patient lacked overt cutaneous or muscular signs on presentation, she later reported joint pain and prior rashes in a distribution typical of DM. While there was no documented clinical evidence of myositis prior to admission, our consulting rheumatologist felt that, given her new-onset severe interstitial lung disease, positive myositis antibody, and reported history of skin rashes involving the forehead, eyebrows, abdomen, upper back, and knuckles, underlying DM was strongly suspected. Additionally, our patient had hyperpigmented, thickened skin over the MCP and PIP joints, consistent with Gottron’s papules. This case emphasizes the value of autoimmune serologies, including extended myositis panels, in evaluating unexplained or rapidly progressive ILD. Early testing can expedite diagnosis and treatment initiation.

Management of MDA5-associated ILD remains challenging. Corticosteroids alone are frequently insufficient, and many patients require combination therapy with additional immunosuppressive agents such as mycophenolate, calcineurin inhibitors, rituximab, or intravenous immunoglobulin (IVIG). Our patient ultimately received rituximab, mycophenolate, and IVIG, consistent with emerging recommendations for aggressive upfront therapy in RP-ILD. Clinical trials have demonstrated favorable outcomes using IVIG in MDA5-associated RP-ILD [16]. In our patient, IVIG was initiated as an adjunct to the existing immunosuppressive regimen at a dose of 2 g/kg divided over two consecutive days. Her clinical status improved significantly after receiving rituximab and IVIG. She was able to be discharged home on 4 L supplemental oxygen.

Case reports support early aggressive treatment for RP-ILD. One described a 38-year-old pregnant woman with new-onset clinically amyopathic dermatomyositis and MDA5 positivity, successfully managed with IV methylprednisolone, cyclophosphamide, and high-flow oxygen without intubation [17]. Additional reports highlight plasma exchange as a rescue therapy in refractory cases after standard immunosuppression failure [18]. This case also underscores a key perioperative teaching point: in post-surgical patients with suspected ARDS and transfusion history, lack of improvement should prompt evaluation for alternative diagnoses. Pulmonologists and Critical Care physicians, often first-line consultants for respiratory failure, play a critical role in recognizing atypical patterns and initiating autoimmune workup. Radiographic evolution, from ground-glass opacities to septal thickening and traction bronchiectasis, can offer early diagnostic clues and guide timely serologic testing.

## Conclusions

This case highlights the diagnostic and therapeutic complexity of anti-MDA5-positive DM with RP-ILD, especially when presenting without classic DM features and mimicking pneumonia or ARDS postoperatively. Persistent hypoxemia and non-resolving pulmonary parenchymal abnormalities should prompt consideration of autoimmune ILD and extended serologic testing. Early initiation of combination immunosuppressive therapy, such as rituximab, mycophenolate, and IVIG, is essential for disease control.
